# Hand Prosthesis with Soft Robotics Technology and Artificial Intelligence for Fine Motor Control

**DOI:** 10.3390/s26051423

**Published:** 2026-02-25

**Authors:** Marco Chaucala-Gualotuña, Danni De la Cruz-Guevara, Johanna Tobar-Quevedo, Maritza Alban-Escobar

**Affiliations:** 1 Department of Energy and Mechanics Sciences, Universidad de las Fuerzas Armadas ESPE, Sangolquí 171103, Ecuador; 2 Department of Electrical, Electronics and Telecommunications Engineering, Universidad de las Fuerzas Armadas ESPE, Sangolquí 171103, Ecuador; 3 Department of Mechanical Engineering, Escuela Politécnica Nacional, Quito 170525, Ecuador

**Keywords:** prosthesis, soft robotic, intelligent sensors, fine motor control

## Abstract

The development of prostheses that accurately reproduce fine motor skills remains a key challenge for daily assistance applications. This research presents the development of a soft robotic hand prosthesis prototype inspired by the natural behavior of muscles and tendons, incorporating internal vacuum-based reinforcement and textured fingertip surfaces to enhance friction and grasp adaptability, without relying on force sensors. The prosthesis reproduces open-hand and tripod pinch movements through myoelectric signals (EMG) acquired via a wearable armband equipped with eight surface electrodes. The signals are processed in real-time and classified by a lightweight dense neural network implemented on a low-power microcontroller. Tendon-driven actuation enables biomimetic motion with smooth and compliant behavior. The proposed system was validated through laboratory-based functional tests using user-specific models, showing response times ranging from 0.49 to 2.00 s and an overall grasping effectiveness of approximately 80% when manipulating small everyday objects with different geometries. These results indicate that the prototype constitutes an accessible and functional solution for fine motor assistance, with potential applicability in low-cost and resource-constrained myoelectric prosthetic systems.

## 1. Introduction

The impairment or functional limitation of an upper limb significantly reduces an individual’s autonomy and quality of life by restricting the execution of daily manipulation and grasping tasks. Consequently, the development of hand prostheses has become an active research field, with numerous proposals exploring diverse mechanical designs and control strategies to partially restore hand functionality. Despite these advances, replicating natural hand motion while maintaining lightweight structures, practical daily usability, and long-term comfort remains an open challenge, particularly when balancing mechanical performance and control simplicity [1,2,3,4].

In this context, soft robotics has emerged as an alternative to conventional rigid robotics by employing elastic materials and compliant structures that enable safer and more adaptive interactions with the environment, which is particularly relevant for hand prostheses that must interact with objects of varying shape and stiffness. Soft robotic hand prostheses can be broadly classified according to their actuation principle and structural configuration. From an actuation perspective, pneumatic and electrically driven systems are predominant, while structurally, designs may be monolithic or rely on motion transmission through tendons or cables. These design choices directly influence grip force, response speed, compactness, durability, and manufacturing complexity, resulting in trade-offs between performance and practical feasibility [5,6,7,8].

Pneumatically actuated soft hand systems commonly rely on PneuNet-type actuators, which generate motion through the pressurization of elastomeric structures with internal cavities. For instance, Ref. [9] reports a soft robotic hand for teleoperation achieving approximately 15 degrees of freedom with response latencies around 0.5 s. Similarly, Ref. [10] presents a rehabilitation-oriented soft hand exoskeleton capable of basic grasp patterns, although its portability is limited by the need for a backpack housing pneumatic pumps and valves. Despite their smooth and compliant motion, recent surveys emphasize that pneumatic systems increase weight, volume, noise, and hardware complexity due to compressed air requirements, limiting their suitability for daily-use hand prostheses [4,11].

Alternatively, soft electronically actuated hand prostheses generate finger motion using electric motors and tendon- or cable-based transmissions, offering greater compactness and portability than pneumatic systems. Early implementations often adopt monolithic architectures, in which flexible joints enable passive finger motion. For example, Ref. [12] presents a monolithic soft hand prosthesis driven by five DC motors, weighing approximately 253 g and producing grasp forces up to 21.5 N, with full finger flexion achieved in about 1.3 s. However, the reported service life of roughly one year highlights structural limitations associated with monolithic soft joints. A pediatric variant of this design is reported in [13], where reduced size and actuator count further trade robustness for simplicity.

To overcome these limitations, tendon-driven soft prostheses integrate compliant elements with cable-based transmission mechanisms to improve mechanical durability and force output. In this context, Ref. [14] presents an optimized hand prosthesis employing five coupled tendons actuated by a single DC motor, achieving grasping forces up to 88 N with a total weight of 290 g. Nevertheless, this design focuses on a limited set of grasps and does not extensively address advanced myoelectric activation strategies.

In parallel, recent advances in embedded machine learning have given rise to the TinyML paradigm, which focuses on deploying machine learning models directly on low-power microcontrollers under strict memory, latency, and energy constraints. This approach has gained increasing relevance in biomedical signal processing, particularly for EMG-based gesture recognition, where real-time response and wearable integration are essential. Prior studies have demonstrated that compact neural networks can achieve competitive accuracy for EMG classification while remaining compatible with resource-constrained embedded platforms [15,16,17].

Although alternative interfaces such as brain–computer interfaces have been explored for prosthetic control, including SSVEP and motor imagery paradigms, these approaches typically require controlled environments, prolonged stimulation, or complex processing pipelines [18,19]. In contrast, EMG-based control remains the most practical solution for daily-use prostheses, enabling high gesture recognition accuracy with relatively simple processing schemes. However, achieving reliable performance on embedded platforms requires careful model simplification and latency-aware design [20,21,22].

Based on this context, it is evident that there remains a need for soft hand prostheses that balance mechanical compliance, structural robustness, aesthetic integration, and low-latency myoelectric control within resource-constrained embedded systems. The main contributions of this study can be summarized as follows: (i) the design of a soft, tendon-driven prosthetic hand that enhances passive grasp stability through textured finger surfaces and internal vacuum-based reinforcement, without relying on additional force or position sensors; (ii) the implementation of a lightweight fully connected neural network (FCNN) optimized for execution on a low-power microcontroller, enabling end-to-end myoelectric control with experimentally measured response times on the order of one second from EMG activation to physical motion; and (iii) an experimental evaluation aligned with the binary deployment of the system (open hand versus tripod pinch), including quantitative analysis of response time, grip success rate, sustained grasp behavior, and false activations during grasping tasks involving everyday objects.

It is important to note that all experimental evaluations were conducted using healthy subjects, and the reported grasping forces and success rates represent preclinical engineering performance metrics. No clinical assessments involving amputee users were performed; therefore, the presented results should be interpreted as a validation of system feasibility and functional stability under controlled conditions, rather than as a direct assessment of clinical efficacy.

## 2. Materials and Methods

This research details the design process of a soft robotics-based prosthesis focused on fine motor skills, the processing of myoelectric signals for gesture recognition, the control of the prosthesis’s movement, and the description of the experimental procedure.

### 2.1. Research Requirements and Scope

Table 1 presents a summary of the main requirements identified and the proposed technical implementation. The requirements described were considered as the basis for guiding each design decision, from the selection of materials to the control architecture.

### 2.2. Prosthesis Development

The prosthesis was designed using computer-aided design (CAD) software (Autodesk Fusion 2605.1.52, Autodesk Inc., USA), as it allows for the model to be edited and adapted according to the user’s morphological characteristics. This subsection presents the development of the CAD model, the selection of materials, and the calculation of energy autonomy.

#### 2.2.1. Mechanical Architecture and CAD Design

For the prosthesis design, finger dimensions were derived from the standardized proportions outlined in DIN 33402-2:2005-12 [23], which offers reference anthropometric dimensions for phalanges, as illustrated in Table 2.

**Figure 1:**
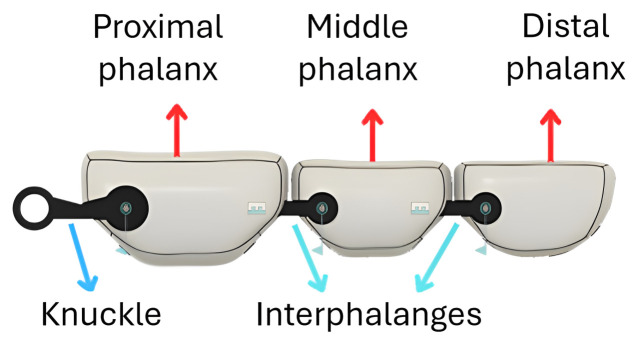
Design of fingers.

The finger structure consists of three segments: proximal, middle, and distal phalanges, while the thumb is composed of two segments: the proximal and distal phalanges. These segments are connected by flexible joints that replicate the function of knuckles and interphalangeal joints, as depicted in Figure 1.

**Figure 2:**
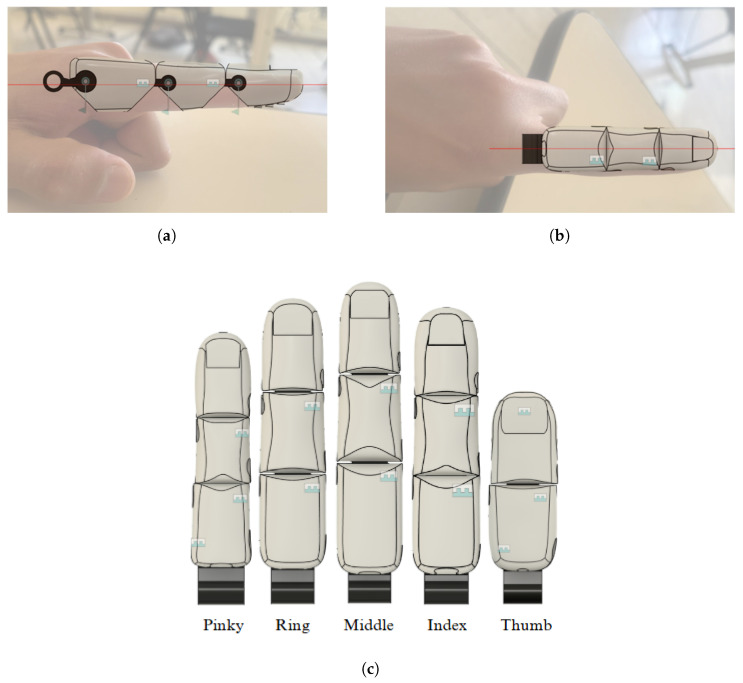
Redesign of the fingers. (**a**) Side view; (**b**) top view; (**c**) redesigned fingers. The red line indicates the central reference axis used during the geometric design.

The resulting visual appearance was unattractive, as it did not account for elements such as finger curvature or nail shape. For this reason, the fingers were redesigned using a real image of a hand as a reference. Based on this, the proportions, shapes, and dimensions were graphically adjusted, resulting in a more realistic design, as shown in Figure 2. In Table 3, it can be seen that the new dimensions are smaller, which can be attributed to the fact that the DIN 33402-2 standard is based on anthropometric data from the European population, whose average proportions tend to be larger than those of the Latin American population. The model used for the redesign is a 27-year-old Ecuadorian man, which reflects regional differences in body structure.

**Figure 3:**
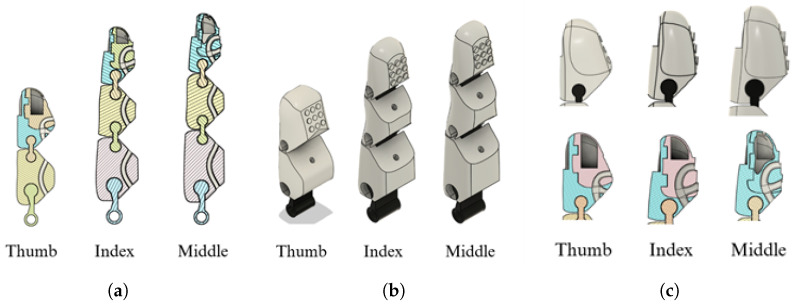
Design of fingers used for fine motor skills: (**a**) channel design for the tension cable, (**b**) fingertip design with rough surfaces to improve the grip area, (**c**) fingertip design with a vacuum capsule for improved grip.

The wire-actuation mechanism draws inspiration from the anatomical structure of the human hand’s muscle-tendon system. In this system, muscles generate force, while tendons transmit it to facilitate joint movement. This biomimetic approach emulates the natural biomechanics of the hand, achieving smoother, more precise, and functional movement trajectories. The prosthesis achieves greater kinematic compatibility and ergonomic behavior during fine motor tasks by replicating this anatomical arrangement. This solution not only improves the prosthesis’s functionality but also achieves a more compact and ergonomic design and adheres to the principles of soft robotics to enable smoother and more efficient movements (Figure 3a). The final result can be seen in Figure 2c; the newly designed fingers improve the aesthetic appearance and aim to be functional for performing fine motor movements.

To improve friction during gripping, rough surfaces were incorporated into the finger contact areas (Figure 3b). To simulate the adaptability of a real finger, internal cavities were designed in the fingertips (Figure 3c) to allow for slight deformation. This approach enables the fingers to conform to objects of different shapes and grasp them without damaging them by applying excessive force, thereby eliminating the need for force or pressure sensors. This feature was implemented in the fingers involved in the tripod pinch grip (thumb, middle, and index).

From a functional standpoint, this controlled deformation of the fingertip acts as a passive feedback mechanism for the user. The mechanical response of the flexible material allows for indirect perception of object stability during grasping, as variations in deformation, contact conditions, and incipient slippage are transmitted through the tendon-driven actuation system and the prosthetic socket. As a result, the applied gripping force is passively self-regulated, reducing the likelihood of excessive force application without the need for dedicated force or pressure sensors.

**Figure 4:**
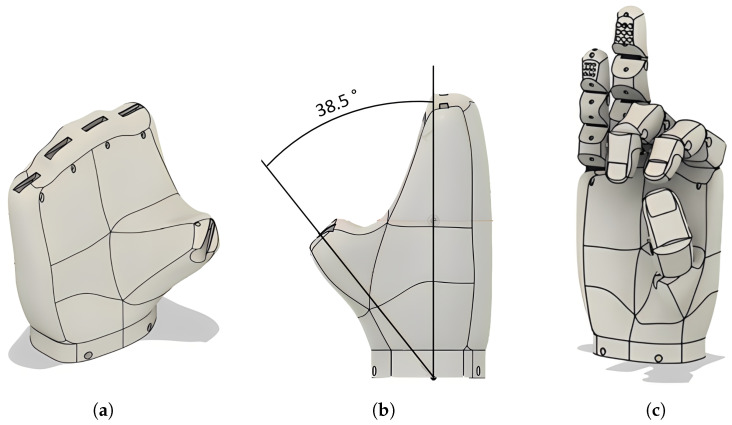
Palm and hand design grip simulation. (**a**) Designed palm; (**b**) angle of inclination; (**c**) grip simulation.

The palm design also takes into account the thumb’s abduction movement, which allows for it to be positioned in the center of the palm and facilitates its interaction with the index and middle fingers. This enables the execution of the tripod pinch grip. The adduction movement returns the thumb to its original position. The selected abduction angle was 38.5°, within the functional range of 35° to 40°, thus ensuring adequate contact between the fingers during grasping (Figure 4).

#### 2.2.2. Materials Selection

3D printing was used to manufacture the prosthesis, owing to its ability to reproduce complex geometries tailored to each user. The design included areas requiring localized flexibility, particularly at the joints and fingertips, which made it necessary to evaluate materials with mechanical properties suitable for these conditions.

The materials were characterized by uniaxial tensile tests on standardized specimens, using different international standards depending on the material type. For TPU, the ASTM D638-14 standard [24] for plastics was applied, whereas for 80A resin, 50A resin, and Ecoflex, the ASTM D412-16 standard [25], recommended for elastomers and rubbers, was used. In these tests, parameters such as ultimate tensile strength and percent elongation were determined, which served as the basis for the final selection (Table 4).

TPU was chosen for the flexible joints, such as the knuckles and interphalangeal joints, because it provides an ideal balance of strength and elasticity. This material allows for controlled deformation that facilitates movement while maintaining structural integrity. Conversely, 50A resin was utilized for manufacturing the fingertips, as its flexibility enables it to mimic the adaptability of human fingertips during contact with objects, while also ensuring it retains its shape after repeated deformations.

#### 2.2.3. Resistance Analysis of Prosthetic Fingers

To evaluate the structural feasibility of the proposed prosthetic hand, a static finite element analysis (FEA) of the index, middle, and thumb fingers was performed. The analysis focused on assessing stress distribution and structural margins under representative loading conditions, considering the actual geometry and materials of the fingers, including TPU for the flexible knuckles, flexible resin (50A) for the fingertips, and PLA for rigid structural components.

The actuation mechanism was not explicitly modeled in the simulation. Instead, external forces were applied at the fingertip regions to represent object contact during grasping. This approach allows for the evaluation of the structural response of the fingers independently of the motor and transmission losses, which were characterized experimentally.

##### Scenario I: Extreme Load Condition

As a conservative validation step, an extreme loading scenario was first analyzed to identify critical stress concentrations and potential failure regions. In this case, a fingertip force of 90 N was applied, corresponding to approximately 70% of the maximum tensile strength of the braided nylon tendon used for actuation. This value was selected as a structural reference derived from the tendon capacity and does not represent a realistic force generated during normal prosthetic operation.

**Figure 5:**
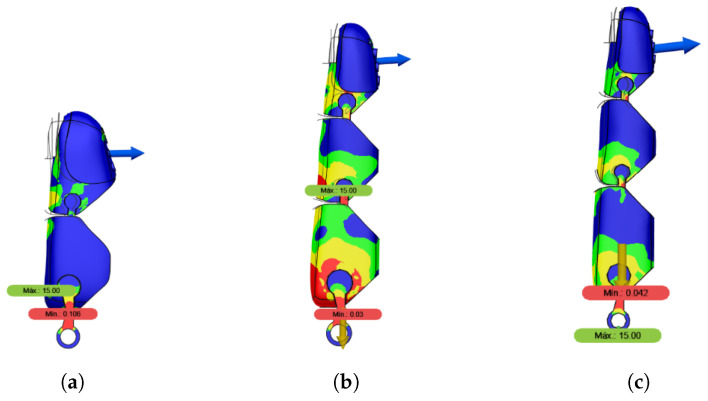
Structural analysis of the prosthetic fingers under extreme load conditions (90 N). (**a**) Thumb; (**b**) index finger; (**c**) middle finger. The color scale represents the von Mises stress distribution, with blue indicating low stress and red indicating high stress; arrows indicate the location of the applied load.

The results of this analysis, illustrated in Figure 5, show that the highest stress concentrations occur at the knuckle regions of the fingers. The minimum safety factors obtained under this extreme load were as follows:

- Index finger: 0.03.
- Middle finger: 0.042.
- Thumb: 0.11.

The safety factors below unity indicate that the applied load exceeds the elastic operating range of the materials. This scenario, therefore, represents a worst-case condition intended solely to highlight critical structural regions and does not correspond to normal operating conditions of the prosthesis.

##### Scenario II: Service Load Based on a Target Safety Factor

To evaluate realistic operating conditions, a second analysis was performed by defining the maximum admissible load for each finger based on a target safety factor of 1.2. This criterion ensures elastic behavior of the materials while accounting for uncertainties in loading and material properties.

**Figure 6:**
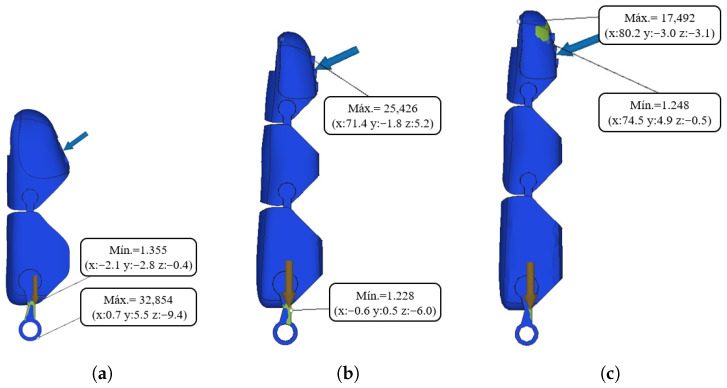
Finite element analysis of the prosthetic fingers under service load conditions defined by a target safety factor of 1.2. (**a**) Thumb; (**b**) index finger; (**c**) middle finger. The color scale represents the von Mises stress distribution, with blue indicating low stress and red indicating high stress; arrows indicate the location of the applied load. Due to the lower applied load, the stress distribution is predominantly in the low-stress range.

In this scenario, the fingertip forces were individually increased for each finger until the minimum safety factor reached the target value. The resulting safety factors are presented in Figure 6. Under this criterion, the middle finger sustained up to 8.2 N, the index finger 8.7 N, and the thumb 10 N. These differences are consistent with the effective length and geometry of each finger. When combined in the tripod pinch configuration, the maximum admissible load is approximately 26.9 N. These values represent the upper bounds of structurally allowable forces for each finger while remaining within the elastic regime.

**Figure 7:**
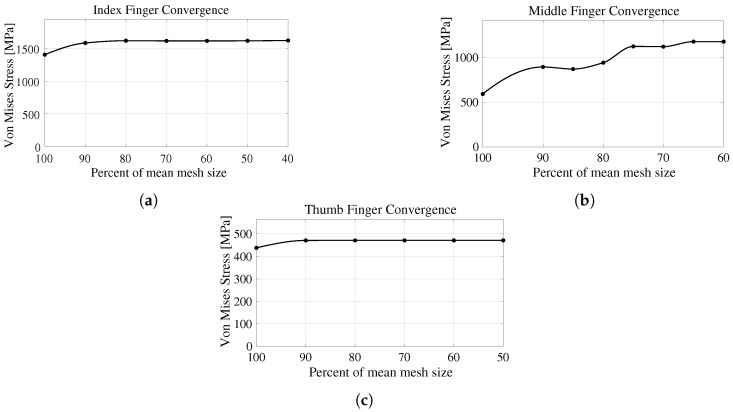
Mesh convergence analysis for the prosthetic fingers. (**a**) Index finger convergence; (**b**) middle finger convergence; (**c**) thumb convergence.

To ensure the numerical reliability of the finite element results, a mesh convergence analysis was performed for the index, middle, and thumb fingers involved in the tripod pinch configuration. The mesh was progressively refined in regions of high stress concentration, particularly around the knuckle joints, until the variation in the maximum von Mises stress between successive refinements was below 1%. This criterion ensures that the reported stress distributions and safety factors are mesh-independent and numerically stable. The convergence behavior obtained for each finger is shown in Figure 7.

#### 2.2.4. Selection of Actuators and Motion Transmission

To evaluate the suitability of different actuators for the proposed soft prosthetic hand, a comparative experimental analysis was conducted using two servo motors with different torque ratings.

The theoretical fingertip force $F_{t}$ was estimated assuming static equilibrium between the motor torque and the tendon force, according to the following:

(1) $$F_{t}=\frac{\tau_{m}}{r}$$

where $\tau_{m}$ is the nominal motor torque and *r* is the effective radius of the pulley or tendon attachment point. This formulation assumes ideal transmission conditions without frictional or elastic losses and therefore represents an upper-bound theoretical estimate.

Initially, an MG90S microservo (TowerPro, Taipei, Taiwan) servo with a nominal torque of 2.2 kg·cm was integrated into the actuation system. Experimental measurements revealed a substantial reduction in the effective fingertip force compared to the theoretical estimate, resulting in limited grasp stability during preliminary tests.

To address this limitation, the actuator was replaced with a higher-torque servo (SDS1901 microservo, Servomy, Shenzhen, China) rated at 8 kg·cm. The same analytical model and experimental procedure were applied to this configuration. Table 5 summarizes the theoretical fingertip forces computed and the experimentally measured forces obtained for both actuators.

The results show that the higher-torque actuator provides a larger effective fingertip force under the same transmission conditions. In both configurations, the difference between theoretical and measured forces is mainly explained by losses inherent to tendon-driven soft robotic systems, such as friction along the tendon path, joint compliance, elastic deformation of soft materials, and non-ideal force transmission. These losses have a greater impact when low-torque actuators are used, reducing the available force at the fingertip. Based on this comparison, the higher-torque servo was selected for the final prototype, as it offers an adequate force margin while maintaining compactness and low system complexity, consistent with the intended use of the prosthesis for fine motor manipulation of lightweight objects.

The tripod pinch configuration, which combines the forces generated by the index and middle fingers against the thumb, produces an effective gripping force ranging from 3.83 to 4.23 N, experimentally validated and shown to be sufficient for the stable manipulation of lightweight everyday objects.

In contrast with the structural analysis presented in the previous section, the experimentally measured fingertip forces obtained with both actuators remain well below the maximum admissible loads defined by the mechanical constraints of the fingers. This indicates that, under the current configuration, the effective gripping force is primarily limited by the actuator torque rather than by structural failure of the finger components. Consequently, the mechanical design exhibits additional load capacity that could be partially exploited by employing actuators with higher torque ratings, provided that such an increase remains consistent with the intended application and does not compromise system size, energy consumption, or control simplicity.

#### 2.2.5. Energy Autonomy

Energy autonomy is a crucial factor in designing functional hand prostheses, as it influences their usability and efficiency in daily activities. In this project, we established an operational autonomy of 3.5 to 4 h, which is adequate for covering partial continuous-use sessions. Future versions may see improvements by integrating higher-capacity, smaller batteries without compromising the system’s ergonomics.

The power consumption of the main system components, including servomotors and the ESP32-S3 control unit (Espressif Systems, Shanghai, China), is presented in Table 6. The total current consumption reaches 741 mA, which allows us to estimate the battery capacity required for the desired operating time.

To determine the required battery capacity, the following formula was used, which considers the system efficiency (η) and the desired operating time (*t*):

(2) $$C=\frac{I\times t}{\eta }$$

Substituting the values: I=741 mA, t=4 h, and η=0.95 (95% efficiency for a new battery), we obtain:

(3) $$C=\frac{741\times 4}{0.95}\approx 3120\;\mathrm{mAh}$$

Based on this consumption, a battery of approximately 3.1 Ah would ensure the desired 4-h runtime under normal operating conditions. This analysis ensures that the prosthesis can operate reliably during everyday use, without overloading the actuators or compromising the user experience.

International standards, such as ISO 13482:2014 [26], establish guidelines for the safety and performance of personal assistance robots, indicating that these devices must be able to operate for reasonable periods without recharging, adapting to the user’s needs.

### 2.3. Processing Myoelectric Signals

**Figure 8:**
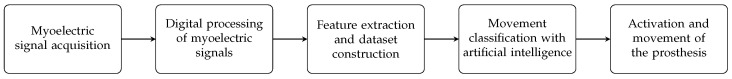
Hand prosthesis control scheme.

The prosthesis control system processes myoelectric (EMG) signals to determine the necessary movements. The overall system flow is illustrated in Figure 8, which details the process from signal acquisition to actuator movement.

#### 2.3.1. Myoelectric Signal Acquisition

EMG signal acquisition was performed using the Myo Armband (Thalmic Labs Inc., Waterloo, Ontario, Canada), which features an array of eight electrodes arranged around the forearm. This device allows for the non-invasive recording of electrical activity generated by muscle contraction. The signals are transmitted wirelessly to the ESP32-S3 microcontroller via Bluetooth communication for subsequent processing.

#### 2.3.2. Digital Processing of Myoelectric Signals

To achieve effective classification, EMG signals must be preprocessed with digital filters that reduce noise and enhance relevant features. A rectification process was employed to eliminate negative values, followed by the application of the filters described in Table 7. The filters used (low-pass, exponential moving average (EMA), and root mean square (RMS)) smooth the signal by attenuating unwanted components.

#### 2.3.3. Feature Extraction and Dataset Construction

The Myo Armband provides eight synchronized EMG channels. After rectification, the signal of each channel is processed using a sliding window of 10 samples to compute the root mean square (RMS) value, which estimates muscle activation amplitude. A first-order low-pass filter with coefficient α=0.1 is then applied, followed by an exponential moving average (EMA) filter with adaptive coefficients.

The EMA coefficient is dynamically adjusted according to the signal amplitude: α=0.2 for high-intensity contractions (RMS > 5000), α=0.05 for low-intensity signals (RMS < 1000), and α=0.1 otherwise.

Each processed sample is represented by an 8-dimensional feature vector:

(4) $$x=[x_{1},x_{2},\ldots ,x_{8}],$$

where $x_{i}$ corresponds to the filtered RMS amplitude of the *i*-th EMG sensor. Each vector is associated with a gesture label corresponding to one of four hand movements.

#### 2.3.4. Movement Classification with Artificial Intelligence

The processed signals are used to train a Fully-Connected Feedforward Neural Network (FCNN) model capable of classifying four types of gestures: open hand, tripod pinch, signal, and fist. The model consists of dense layers with ReLU activation functions, batch normalization, and dropout regularization.

Each layer performs transformations of the form:

(5) $$h^{\left(l\right)}=\phi \left(W^{\left(l\right)}h^{\left(l-1\right)}+b^{\left(l\right)}\right),$$

where

- $h^{\left(l\right)}$ is the output vector of layer *l*.
- $W^{\left(l\right)}$ is the weight matrix connecting layer l−1 to layer *l*.
- $h^{\left(l-1\right)}$ is the output of the previous layer (l−1).
- $b^{\left(l\right)}$ is the bias vector for layer *l*.
- ϕ is the nonlinear activation function, which in this case is ReLU.

The activation function used is ReLU (Rectified Linear Unit), defined as

(6) ReLU(x)=max(0,x),

Which only allows for positive values to be passed to the next layer. This function helps the network learn nonlinear relationships between the data and facilitates gradient propagation during training, [27]. To improve the model’s stability and generalization ability, two additional techniques are incorporated. The first is batch normalization, which adjusts the activations of each layer to have zero mean and unit variance:

(7) $$\mathrm{BN}\left(x\right)=\frac{x-\mu }{\sigma },$$

where μ and σ are the mean and standard deviation computed on the current training batch [28]. The second technique is dropout, which involves randomly deactivating a percentage of the neurons during training. This is represented as

(8) $$\mathrm{Dropout}\left(x,p\right)=\left\{\begin{array}{cc} 0 & \mathrm{with}\;\mathrm{probability}\;p,\\ x & \mathrm{with}\;\mathrm{probability}\;1-p, \end{array}\right.$$

where *p* is the probability of deactivating a neuron. In this model, a value of *p* = 0.75 is used [29]. The output layer produces a vector $z\in R^{4}$, whose components represent the evidence in favor of each class. This vector is transformed into probabilities using the softmax function:

(9) $$\mathrm{softmax}\left(z_{i}\right)=\frac{e^{z_{i}}}{\sum_{j=1}^{K}e^{z_{j}}},\;i=1,2,\ldots ,K,$$

where

- $z_{i}$ is the raw output (logit) for class *i*.
- *K* is the total number of classes (in this case, K=4).
- $e^{z_{i}}$ is the exponential of $z_{i}$.

The softmax function converts these values into a probability distribution, useful for classification tasks [30]. During training, a loss function is employed to measure how far the model’s predictions are from the actual classes. Sparse categorical crossentropy is used, which is suitable for integer labels:

(10) $$L=-\log({\hat{y}}_{c}),$$

where

- ${\hat{y}}_{c}$ is the probability that the model assigns to the correct class *c*.
- *L* is the loss (error) value for a given sample.

This value is minimized during training to improve the model’s performance [31]. Optimization is carried out using the Adam algorithm, which is a gradient descent method that automatically adjusts the learning rate for each parameter by utilizing moving averages of the gradients:

(11) $$\theta_{t+1}=\theta_{t}-\alpha \cdot \frac{{\hat{m}}_{t}}{\sqrt{{\hat{v}}_{t}}+\epsilon },$$

where

- $\theta_{t}$ is the parameter value at step *t*.
- α is the learning rate.
- ${\hat{m}}_{t}$ and ${\hat{v}}_{t}$ are the estimates of the first and second moments of the gradient.
- ϵ is a small value to prevent division by zero.

This optimizer enables faster and more stable training, especially in deep networks [32]. The neural network was implemented and trained using the TensorFlow/Keras framework (TensorFlow version 2.18.0, Google LLC, USA). The dataset was randomly shuffled and divided into training (60%), validation (20%), and test (20%) subsets. Training was performed for a maximum of 75 epochs with a batch size of 32.

Early stopping was applied by monitoring the validation loss with a patience of 10 epochs, and a learning rate reduction strategy was used to improve convergence. All hyperparameters were fixed throughout the experiments to ensure reproducibility.

**Figure 9:**
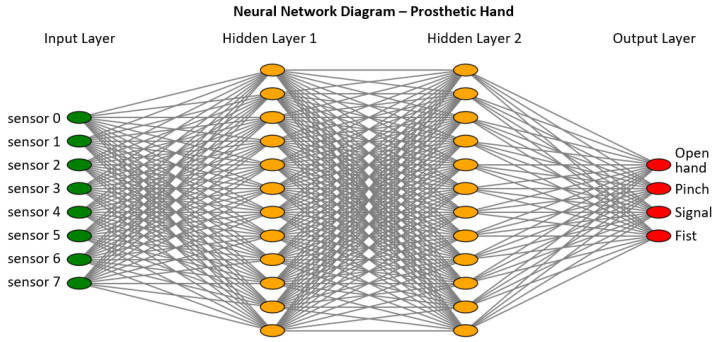
Neuronal network diagram.

Overall, the implemented model is an FCNN with two hidden layers of 12 neurons each, ReLU activation, batch normalization, and dropout, followed by a softmax output layer for four-class classification. Its design allows for the efficient processing of EMG signals and the classification of different types of hand movements. Table 8 specifies the values used to construct the neural network (Figure 9).

#### 2.3.5. Activation and Movement of the Prosthesis

The activation of the prosthesis is based on predicting the class of the EMG signal generated by the previously trained model. Once the signal is captured, it is digitally processed and classified to determine which of the predefined hand gestures it most likely corresponds to. Although the classification model is capable of recognizing four gestures (open hand, tripod pinch, fist, and point), only the open-hand and tripod pinch movements were implemented in the prosthetic controller. The remaining gestures were used exclusively to increase the robustness and generalization capability of the model during the training stage.

**Figure 10:**
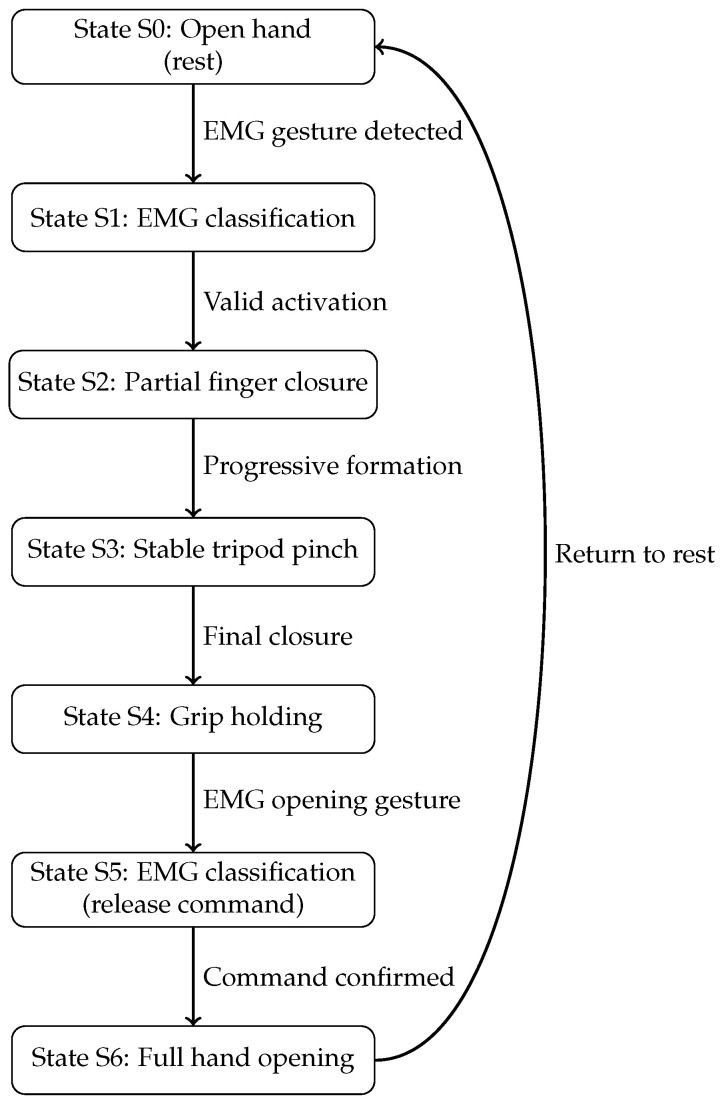
State-machine diagram of the prosthetic movement sequence during the tripod pinch grip.

Based on the classification outcome, the prosthetic system operates according to a state-machine-based control strategy that governs the execution and sequencing of movements. This approach allows for the complex behavior of the prosthesis to be decomposed into a set of discrete functional states, each representing a specific phase of the movement. To provide a clear and intuitive visualization of this behavior, a dynamic schematic diagram of the prosthetic movement sequence is presented in Figure 10.

The movement sequence begins in an open-hand resting state. Upon detection and validation of an EMG gesture corresponding to a grasp command, the controller transitions to a partial finger closure state, followed by a progressive formation of the tripod pinch. Once the desired grasp configuration is achieved, the prosthesis enters a grip-holding state, maintaining the applied force until an EMG opening command is detected. After confirmation of the release command, the system performs a full hand opening and returns to the resting state, completing the movement cycle.

If the predicted EMG class does not correspond to one of the enabled movements, the prosthesis retains the last valid gesture executed. This behavior acts as a safety mechanism against transient misclassification or signal interruptions, ensuring stable operation and preventing unintended motions. The user can resume normal control by repeating a recognized EMG gesture, allowing for the system to continue operating reliably under real-world conditions.

## 3. Experiments and Results

Performance metrics were evaluated on a per-subject and per-session basis. These metrics included classification accuracy, average response time, sustained grasp duration evaluated over four trials, and repeated grasping performance based on 20 trials per object.

For the performance evaluation of the prosthesis, the analysis was divided into three stages: (1) EMG signal acquisition and response, (2) signal processing and classification, and (3) actuation and control of the prosthesis.

### 3.1. Description of the Experimental Procedure

The experimental evaluation was conducted in a controlled laboratory environment with the participation of ten healthy volunteers. Subjects were selected based on availability criteria and had no upper-limb amputations or known neuromuscular disorders. To ensure homogeneous experimental conditions, all participants were right-handed and performed the experiments using the same upper limb.

**Figure 11:**
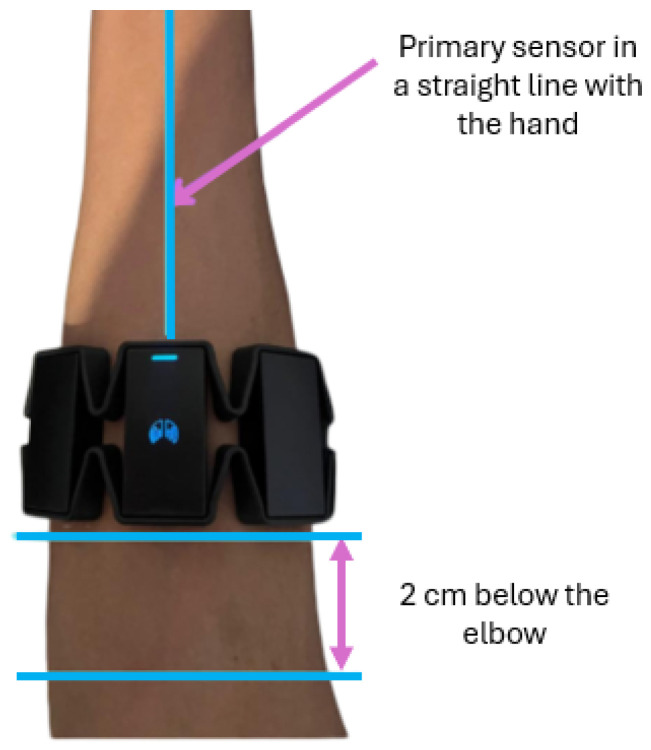
Myo Armband location for signal capture and prosthesis control.

Myoelectric signals were acquired using a wearable EMG armband positioned on the right forearm, approximately 2 cm below the elbow, while maintaining a consistent orientation of the main reference sensor, as illustrated in Figure 11. Before data acquisition, participants received standardized instructions outlining the experimental protocol and the execution of gestures. All tests were performed with the participants seated in the same position to reduce posture-related variability.

Table 9 summarizes the demographic and anthropometric characteristics of the participants involved in the experimental procedure.

Each participant executed four predefined gestures: open hand, tripod pinch, pointing, and fist. For each gesture, approximately 1250 myoelectric signal samples were recorded using a fixed acquisition window of 50 ms. These data, acquired in a single experimental session per participant under controlled conditions and with short rest intervals between gesture executions, were used exclusively for the analyses presented in Section 3.2 and Section 3.3, which focus on EMG signal acquisition, inter-user variability, and classification performance.

In contrast, additional functional evaluations—including response time, sustained grasp duration, and object grasping tests—were conducted exclusively with user E10. This subject was selected due to stable signal behavior and greater availability, enabling extended testing sessions aimed at validating the functional performance of the prosthetic system, as described in Section 3.4. For all experimental evaluations, the prosthesis was secured on a stable surface and operated without direct physical attachment to the user, ensuring controlled and repeatable testing conditions.

### 3.2. EMG Signal Acquisition and Response

This subsection focuses on the analysis of EMG signal characteristics across multiple users and on the selection of an appropriate analysis window for real-time gesture classification. The experiments were conducted in a single session per participant on the same day, under controlled conditions, and following the acquisition protocol described in the previous subsection.

**Figure 12:**
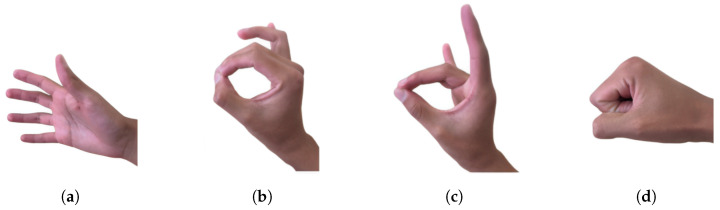
Hand movements for signal capture. (**a**) Open Hand; (**b**) tripod pinch; (**c**) signal; (**d**) fist.

During each session, participants performed a predefined sequence of gestures: open hand, tripod pinch, point, and fist (Figure 12). The myoelectric activity captured by the armband was acquired using the corresponding software interface and stored for offline analysis and model validation.

To analyze the effect of the temporal window length on signal quality and classification performance, EMG data were acquired using different window sizes (100 ms, 50 ms, and 30 ms) under identical experimental conditions. In all cases, the signals were pre-filtered according to the parameters defined in the methodology. The resulting waveforms are illustrated in Figure 12. As reported in the literature, the selection of the analysis window involves a trade-off between temporal resolution and signal stability, where shorter windows reduce latency but increase variability, while longer windows provide smoother signals at the expense of slower response [33].

**Figure 13:**
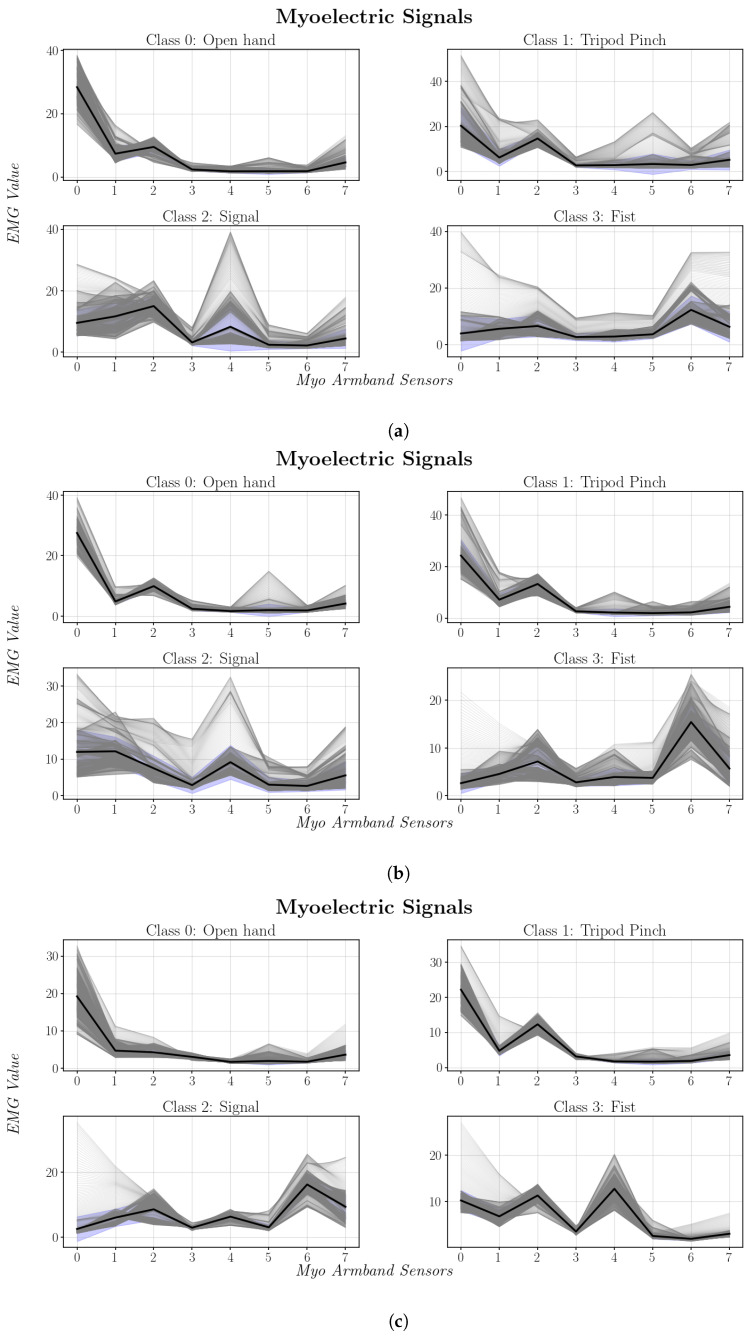
Myoelectric signal reception. (**a**) EMG signals for sampling time of 30 ms; (**b**) EMG signals for sampling time of 50 ms; (**c**) EMG signals for sampling time of 100 ms. Darker regions indicate areas where multiple EMG signal traces overlap.

The results show that the sampling time has a direct effect on the morphology and clarity of the recorded signal. At 100 ms (Figure 13c), the signal exhibits higher amplitude but reduced temporal resolution, which may introduce ambiguity in the classification stage. In contrast, a 30 ms window (Figure 13a) is more sensitive to small muscle variations, resulting in faster response but increased instability and lower amplitude. The 50 ms window (Figure 13b) provides a balance between temporal resolution and signal stability, enabling reliable classification without significantly increasing latency. For this reason, a 50 ms window was selected for subsequent experiments.

**Figure 14:**
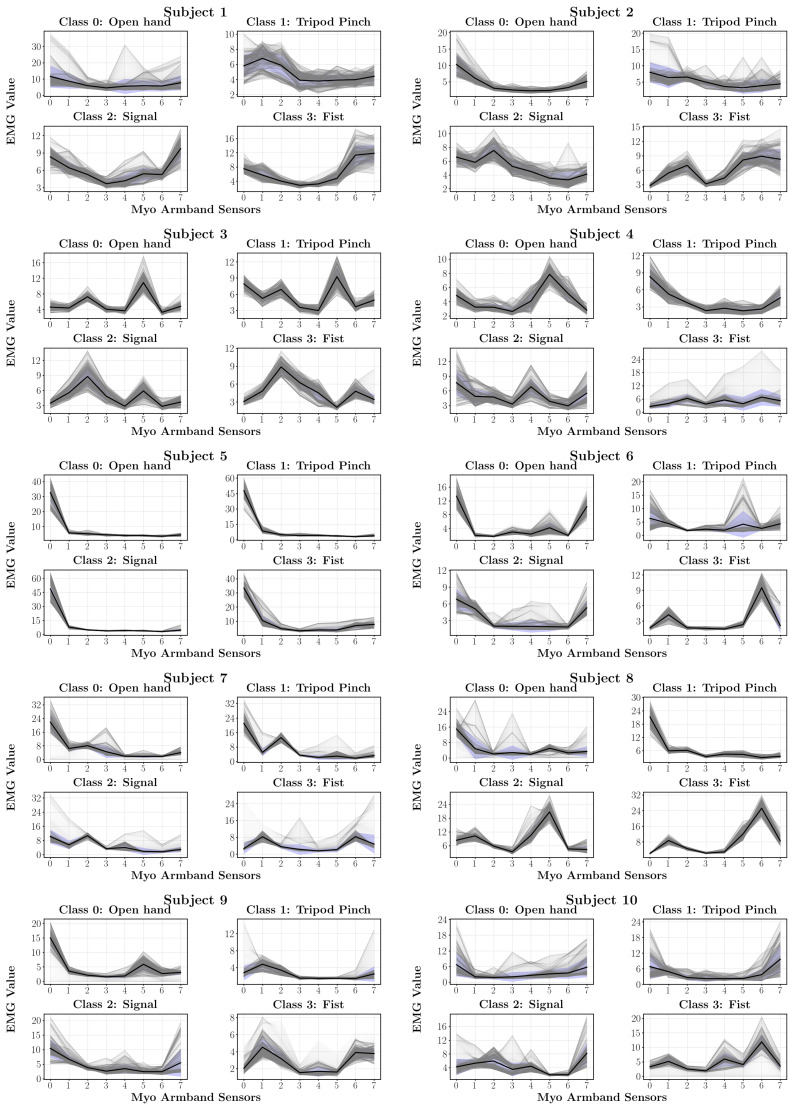
Myoelectric signal patterns captured from the user sample. Darker regions indicate areas where multiple EMG signal traces overlap.

Inter-user variability in myoelectric activation patterns was observed, as EMG signals recorded from different users performing the same gestures showed notable differences in waveform shape, amplitude, and dominant channels (Figure 14). This behavior, widely reported in the literature, is attributed to individual anatomical and physiological factors that limit the generalizability of surface EMG signals [34,35].

These observations indicate that the proposed system cannot rely on a fully generalized classification model without a significant degradation in performance. Instead, reliable control requires adapting the model to the specific myoelectric characteristics of each user. Consequently, the system was designed following a user-oriented approach, in which the signal processing pipeline and network architecture remain fixed, while the model parameters are trained individually.

User E10 was selected for extended functional evaluation due to the stability and repeatability of the EMG signals observed during continuous prosthetic control. Nevertheless, the same training and calibration procedure can be applied to other users, enabling personalization without modifying the underlying control framework.

### 3.3. Signal Processing and Classification

Based on the inter-user variability observed, individual machine learning models were trained for each participant using the same signal processing pipeline and network architecture. All models were trained using data acquired during the same experimental session. Table 10 summarizes the main training and validation metrics obtained for each user-specific model.

The following accuracy ranges were considered as criteria for evaluating the models:

- <75%: Unsatisfactory model.
- 80–95%: Well-trained model.
- 95%: Possible overfitting.

Models corresponding to users E4, E8, E9, and E10 achieved accuracies above 95%, which could indicate potential overfitting. Overfitting occurs when a model memorizes subject-specific patterns rather than learning generalizable features. However, high accuracy does not necessarily imply poor model behavior in user-specific EMG control, where the objective is reliable recognition of signals from the same user.

In particular, user E8 represents an atypical case within the experimental set. Although the model reached 100% accuracy, subsequent tests confirmed stable performance and correct gesture recognition for new signals from the same user. This behavior suggests that the high performance is primarily attributable to the clarity and consistency of the recorded EMG signals rather than to model overfitting.

### 3.4. Actuation and Control of the Prosthesis

**Figure 15:**
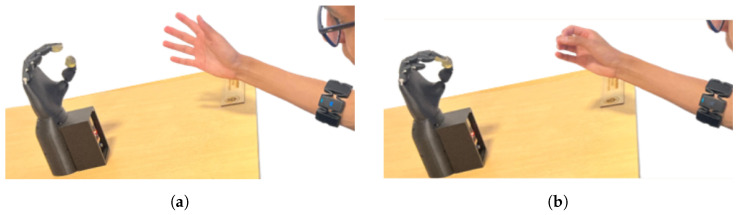
Prosthesis movement tests with the user E10. (**a**) Open hand; (**b**) tripod pinch.

Once the training process was completed, the prosthesis was powered on, and the corresponding code was loaded. For this validation phase, the prosthesis was secured in place on a stable surface, without direct contact with the user, to conduct controlled system tests. Figure 15 shows the activation of the prosthesis by user E10.

The experimental tests were designed from a functional and engineering-oriented perspective to validate the integration between EMG signal acquisition, gesture classification, and mechanical actuation. Since the proposed system corresponds to a laboratory prototype, the evaluation does not claim clinical validation. Instead, the tests focus on verifying gesture detection reliability, response time, and grasp stability under controlled and reproducible conditions.

#### 3.4.1. Response Time

To evaluate the dynamic performance of the controller, experimental tests were conducted focusing on the response time to EMG-driven gesture transitions. These tests were performed using the model corresponding to subject E10, selected for the functional evaluation stage described in the previous subsection.

The model recognizes four gestures (open hand, tripod pinch, pointing, and fist); however, only two of them (open hand and tripod pinch) are functionally replicated by the prosthesis. Table 11 reports the activation times measured during consecutive transitions between these two functional gestures. The average activation time was 1.14 s for transitions from open hand to pinch and 1.02 s for transitions from pinch to open hand, resulting in an overall mean response time of 1.05 s.

The measured response times indicate that the system is capable of reliably detecting gestures and executing the corresponding movements with limited latency. Although response time is influenced by subject-specific EMG characteristics, the obtained results demonstrate effective integration between EMG signal acquisition, gesture classification, and mechanical actuation under controlled conditions.

#### 3.4.2. Duration in a Fixed Grasp Position

This experiment aimed to evaluate the ability of the prosthesis to maintain a stable grasp configuration over a prolonged period, focusing on the tripod pinch gesture. This assessment is relevant because sustained muscle contraction may induce user fatigue, progressively altering EMG signal characteristics and affecting the stability of myoelectric control.

The experimental protocol required the user to execute the tripod pinch gesture starting from a fully open-hand configuration. The holding time was measured from the moment the prosthesis fully reached the pinch position. The user was then instructed to maintain the contraction for as long as possible until a noticeable variation in the EMG signal led to grasp loss or a transition to a different class. For consistency, each trial started from the same initial hand configuration.

Table 12 presents the holding times obtained using a marker-type object, selected as a representative case due to its stable geometry and low weight. The first four trials were conducted during a single experimental session, while the remaining seven trials were performed during a separate session on a different day. A rest period of approximately 30 s was introduced between consecutive trials to limit short-term muscle fatigue.

The results indicate that, in successful trials, the prosthesis was able to maintain the tripod pinch grasp for an average duration of 17.92 s before fatigue-related variations in the EMG signal occurred. This behavior highlights an inherent limitation of EMG-based control systems, in which grasp stability depends on the user’s ability to sustain muscle activation.

Despite this limitation, the obtained holding times demonstrate that the system can maintain a functional grasp position for sufficiently long intervals to perform basic fine manipulation tasks. These results complement the response time evaluation, showing that the system provides not only timely gesture activation but also adequate grasp stability under sustained use conditions.

#### 3.4.3. Grip Tests

This experimental test aimed to evaluate the prosthesis’s ability to grasp small and lightweight objects using a tripod pinch under controlled and repeatable conditions. Three objects with distinct geometries were selected: a marker, a lipstick, and an eraser. These objects differ in shape, size, and surface texture, allowing for the assessment of grasp adaptability and stability under varying conditions.

All grasping trials were conducted under consistent initial conditions. At the beginning of each attempt, the prosthesis was initialized in a fully open-hand position. Each trial was triggered by the detection of the EMG signal corresponding to the tripod pinch gesture, ensuring consistent activation across tests.

A grasp was defined as successful when the object was lifted and held in a stable position for a continuous interval exceeding 12 s. Conversely, a trial was considered unsuccessful if the object could not be lifted or was released during execution. For each object, 20 independent trials were performed. A rest period of approximately 30 s was introduced between consecutive trials, while a longer rest interval of approximately 2 min was applied when switching between different objects to limit muscle fatigue.

**Figure 16:**
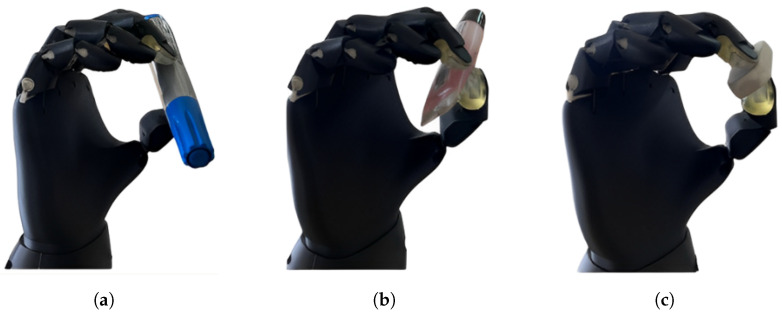
Representative grip tests performed using a tripod pinch on objects with different geometries under controlled experimental conditions. (**a**) Grip test with a marker; (**b**) grip test with a lipstick; (**c**) grip test with an eraser.

Representative grasping trials performed under the defined experimental conditions are illustrated in Figure 16. The quantitative results of the repeated grasping experiments are summarized in Table 13. On average, the prosthesis achieved 16 successful grasps out of 20 attempts per object, corresponding to an overall effectiveness of approximately 80%.

The grasp trajectory and applied force remained consistent across repeated trials, as the control system generates predefined and repeatable activation patterns for each functional gesture. This behavior is supported by prior dynamometer-based measurements conducted on the fingers involved in the tripod pinch, confirming that the selected actuators provide sufficient and repeatable force to securely grasp lightweight objects.

Overall, the results demonstrate reproducible grasping behavior and stable functional performance, validating the prosthesis’s capability to reliably perform basic fine motor tasks under controlled conditions.

## 4. Discussion of Results

This study’s results indicate that effective performance in myoelectric hand prosthetics can be attained without dependence on costly hardware, many sensing modalities, or complex algorithms. Unlike various contemporary methods that prioritize intricate feature extraction processes or resource-intensive deep architectures [20,21,22], the proposed system achieves dependable performance utilizing readily available components and a compact neural network executed on a low-power microcontroller, which is especially pertinent for embedded and assistive applications.

In contrast to studies like [20], which integrate handcrafted feature extraction methods (such as RMS) with adaptive systems like ANFIS, the current research successfully classifies gestures using brief analysis windows of 50 ms, while sustaining average end-to-end response times near 1 s. This trade-off provides a pragmatic benefit for real-time prosthetic control, particularly in contexts where low latency, restricted computational resources, and energy efficiency are essential limitations.

The analysis of EMG signals using machine learning highlighted the necessity of user-specific model training due to pronounced inter-individual variability. Factors such as forearm morphology, muscle activation patterns, and prior user experience significantly influence EMG signal characteristics. This observation contrasts with paradigms such as steady-state visual evoked potential (SSVEP)-based interfaces, where activation signals can be detected using subject-independent mathematical formulations [36]. In contrast to works where EMG electrodes are manually positioned and additional variability between sessions is reported [12,37], the use of a wearable armband and a standardized acquisition protocol helps reduce placement-related variability. In the present work, functional performance metrics were therefore evaluated under a personalized deployment scenario, reflecting realistic conditions for myoelectric prosthesis use rather than population-level generalization. Consequently, the results indicate that cross-user model standardization is not feasible under the evaluated conditions, and that reliable performance requires an individualized training process.

From a mechanical perspective, the soft robotics-based design inspired by the behavior of human muscles and tendons enabled smooth and natural execution of fine motor actions, such as the tripod grasp, which are typically associated with the manipulation of lightweight and small-scale everyday objects. Unlike approaches that incorporate force, current, or strain gauge sensors to enhance feedback [12,38], the proposed design relies on passive mechanical compliance achieved through tendon-driven actuation, textured fingertip surfaces, and internal vacuum-based reinforcement.

This design choice reduces system complexity and cost while maintaining sufficient grasp stability for fine motor tasks, making the prosthesis particularly suitable for assistive applications and resource-constrained contexts. However, this approach is not intended for tasks involving heavy objects or high gripping forces.

Although the mechanical analyses indicate that the prosthetic structure can withstand a load of 26.9 N when considering the combined resistance of the fingers in the tripod pinch configuration, the effective gripping force is ultimately limited by the actuator torque. Experimental evaluations revealed that, due to transmission losses and the selected motor characteristics, the maximum force actually achieved by the prosthesis during the tripod pinch gesture is approximately 3.83 to 4.23 N. This confirms that, in the current prototype, the operational force range is constrained by the actuation system rather than by the structural resistance of the fingers.

Regarding geometric and aesthetic considerations, the prosthesis design initially followed the anthropometric proportions defined by the DIN 33402 standard [23]. These dimensions were subsequently adjusted based on real anthropometric measurements, allowing for the morphology to better reflect characteristics representative of the target user population. This process highlighted morphogeometric differences between populations and underscores the importance of regional adaptation in prosthetic design. The resulting prosthesis achieves a balanced integration of functionality, structural simplicity, and visual realism, including details such as palm curvature, fingernails, and fingertip geometry, which are important factors for user acceptance.

The combination of flexible materials and tendon-driven actuation proved advantageous when contrasted with pneumatically actuated soft hands, such as those reported in [9,39]. These pneumatic systems demonstrate a high level of compliance and the ability to generate smooth, adaptive motions, particularly in applications requiring large deformation or teleoperation. However, the proposed prosthesis achieved a more compact, quiet, and visually discreet configuration, which facilitated stable grasp execution during laboratory tests and favors its integration into everyday environments.

In comparison with highly biomimetic hand designs that employ complex skeletal structures and tendon routing to maximize anatomical fidelity [37], the present approach adopts a simplified mechanical and control architecture. While such biomimetic models offer enhanced dexterity and closer resemblance to the human hand, the results obtained in this study indicate that reliable and repeatable performance for fine motor tasks can be achieved using a reduced number of components, prioritizing energy efficiency, ease of replication, and embedded feasibility.

Functional validation demonstrated the combined performance of the proposed control strategy and mechanical structure. User-specific classification models achieved accuracies above 82.5% in most cases, with an overall mean accuracy of 87.94%, despite pronounced inter-user variability. Functional gesture transitions exhibited response times ranging from 0.49 to 2.00 s, comparable to those reported for EMG-driven soft prosthetic systems, such as the 1.3 s finger flexion time reported in [12] and the 3.3 s reported in [37]. Sustained grasp experiments yielded an average holding time of 17.92 s, indicating stable control under prolonged muscle activation, while repeated grasping trials with everyday objects achieved an overall effectiveness of approximately 80%. Together, these results confirm that the proposed system can reliably support basic fine manipulation tasks under controlled conditions.

The inclusion of rough fingertip surfaces and internal vacuum-based reinforcement contributed to improved grip stability without requiring pressure sensors or haptic feedback systems. These results highlight how passive mechanical features can complement simplified control strategies, supporting functional performance while preserving a low-cost and user-centered design.

## 5. Conclusions

This research presents the design and validation of a functional, accessible, and visually realistic myoelectric hand prosthesis aimed at supporting fine motor tasks. The proposed system successfully integrates soft robotic principles with a tendon-driven actuation mechanism and a lightweight EMG-based control strategy, enabling reliable execution of open hand and tripod pinch movements in real-time.

The myoelectric control scheme demonstrated robust gesture recognition using EMG signals acquired through a wearable armband equipped with eight electrodes and processed by a fully connected neural network optimized for execution on a low-power microcontroller. Classification accuracies above 82.5% were obtained in most cases, with some users achieving values close to 100%, confirming the feasibility of accurate and low-latency embedded EMG control for prosthetic applications.

From a mechanical perspective, the soft, tendon-driven design enabled smooth and adaptive motion without relying on additional force, position, or current sensors. This structural simplification reduces system complexity and cost while maintaining functional stability. Furthermore, the geometric design, based on DIN 33402 anthropometric proportions and adjusted using measurements from a real hand, contributed to an improved aesthetic appearance, which is an important factor for user acceptance in daily-use prostheses.

Experimental validation under functional conditions showed an average response time of 1.05 s and an effectiveness of approximately 80% when grasping small everyday objects with different geometries. These results demonstrate the practical applicability of the proposed prototype for assistive and rehabilitation-oriented scenarios, while also highlighting its potential as a low-cost and replicable solution.

As future work, it is proposed to expand the range of movements reproduced by the prosthesis by incorporating additional degrees of freedom in the thumb. This extension would enable a broader set of grasp patterns and increase the functional versatility of the system while preserving its current mechanical simplicity and control architecture. 

## Figures and Tables

**Table 1:** Functional requirements and implementation techniques for myoelectric hand prostheses for fine motor skills.

Functional Requirement	Technical Implementation
Realistic prosthesis design based on human dimensions	Application of DIN 33402-2:2005-12 standard [23] supplemented with anthropometric adjustments obtained from actual hand dimensions.
Natural movement inspired by muscle biomechanics	Use of tension wires that simulate the action of tendons and muscles, allowing for biomimetic flexion and extension of the fingers.
Resistant and adaptable structure for manipulating small objects of various geometries	Integration of rigid and flexible materials under the principles of soft robotics, ensuring structural stability and adaptability in gripping.
Improved friction and adaptability in contact with objects	Incorporation of internal vacuum capsules and rough surfaces on the fingertips, increasing grip effectiveness without the need for force sensors.
Real-time control of the prosthesis	Acquisition of EMG signals using surface electrodes, processed with a machine learning algorithm, achieving a minimum reliability of 80%.

**Table 2:** Finger sizing according to DIN 33402.

Finger	Proximal Phalanx (mm)	Middle Phalanx (mm)	Distal Phalanx (mm)
Thumb	45–55	N/A	28–35
Index	50–60	30–40	20–25
Middle	55–65	35–45	22–28
Ring	50–60	30–40	20–25
Little	40–50	25–35	18–22

Note: N/A indicates that the thumb does not have a middle phalanx.

**Table 3:** Dimensioning of fingers selected for the fine motor prosthesis.

Finger	Proximal (mm)	Middle (mm)	Distal (mm)
Thumb	27	N/A	27
Index	27	22	23
Middle	32	25	25
Ring	27	23	25
Little	22	17	21

Note: N/A indicates that the thumb does not have a middle phalanx.

**Table 4:** Mechanical properties of soft materials used in finger design.

Material	Ultimate Stress (MPa)	Elongation % (u)	Elongation % (Gage)	Observation
TPU	31.29	466.02	372.22	High mechanical strength and good flexibility.
Resin 50A	1.84	82.79	100	Low strength, but adequate flexibility for contact zones.
Resin 80A	3.99	90.98	80	Higher strength than 50A, but less flexible.
Ecoflex	0.77	645.30	620	Extremely flexible, but very low strength.

**Table 5:** Comparison between theoretical and measured fingertip forces for different servomotors.

Finger	Length (m)	Theoretical Force (MG90S) [N]	Measured Force (MG90S) [N]	Theoretical Force (SDS1901) [N]	Measured Force (SDS1901) [N]
Thumb	0.130	1.66	0.89	6.04	2.22
Index	0.210	1.03	0.89	3.74	1.78
Middle	0.225	0.96	0.89	3.49	1.78

**Table 6:** Electric power consumption of the control system elements and actuators.

Element	Current (mA)	Voltage (V)	Torque (kg·cm)
MG90S Servo	100	7.4	2.2
MG90S Servo	100	7.4	2.2
9kg-cm Servo	150	7.4	8.0
9kg-cm Servo	150	7.4	8.0
9kg-cm Servo	150	7.4	8.0
ESP32 S3	91	5.0	0.0
Total Consumption	741		

**Table 7:** Digital filters used for EMG signal processing.

Filter	Parameter	Description
Low-pass filter	α=0.1	First-order IIR low-pass filter applied to the RMS signal to attenuate high-frequency noise.
EMA filter (default)	α=0.1	Exponential moving average coefficient used under normal signal conditions.
EMA filter (high signal)	α=0.2	EMA coefficient applied when RMS amplitude exceeds 5000, increasing responsiveness to strong contractions.
EMA filter (low signal)	α=0.05	EMA coefficient applied when RMS amplitude is below 1000, providing smoother filtering for weak signals.
RMS filter	N=10	Root mean square computed over a sliding window of 10 samples to estimate muscle activation amplitude.

**Table 8:** Neural network architecture and training hyperparameters.

Component	Description
Input	8 features from EMG sensors
Dense Layer 1	12 neurons, ReLU activation function
Batch Normalization	Normalizes activations for training stability
Dropout 1	Dropout rate of 75% (0.75)
Dense Layer 2	12 neurons, ReLU activation function
Batch Normalization	Second batch normalization
Dropout 2	Dropout rate of 75% (0.75)
Output Layer	4 neurons, Softmax activation function
Loss Function	Sparse categorical crossentropy
Optimizer	Adam (learning_rate = 0.006)
Batch Size	32 samples per batch
Epochs	75
Early Stopping	Patience = 10 epochs (validation loss)
Weight Initialization	Default Keras initialization (Glorot uniform)

**Table 9:** Characteristics of the experimental subjects.

Subject	Gender	Age	Handedness	Forearm Circumference (mm)
E1	Female	19	Right-handed	64
E2	Female	20	Right-handed	78
E3	Female	23	Right-handed	85
E4	Female	24	Right-handed	81
E5	Female	20	Right-handed	70
E6	Male	25	Right-handed	68
E7	Male	27	Right-handed	75
E8	Male	31	Right-handed	82
E9	Male	27	Right-handed	80
E10	Male	25	Right-handed	68

**Table 10:** Training results for established users.

Model	Test Accuracy	Training Time (s)	Training Accuracy	Validation Accuracy	Training Loss	Validation Loss
E1	82.50%	17.77	83.50%	84.30%	0.4772	0.4806
E2	83.90%	11.26	86.43%	85.60%	0.3341	0.3426
E3	91.10%	8.69	89.57%	88.80%	0.5136	0.5222
E4	100.00%	13.16	100.00%	100.00%	0.0140	0.0143
E5	74.20%	7.21	76.80%	76.40%	0.4617	0.4791
E6	72.20%	8.14	75.93%	76.10%	0.3492	0.3533
E7	79.50%	6.91	80.43%	80.60%	0.4399	0.4555
E8	100.00%	11.75	100.00%	100.00%	0.0092	0.0097
E9	98.50%	13.23	98.57%	99.10%	0.1601	0.1623
E10	97.50%	11.80	98.20%	97.90%	0.1789	0.1779
Mean	87.94%	10.99	88.94%	88.88%	–	–
Std. Dev.	10.84%	3.36	9.71%	9.73%	–	–

**Table 11:** Execution times recorded for hand-to-pinch and pinch-to-hand movements of the prosthesis.

Sample	Executed Action	Activation Time (s)
1	Hand to Pinch	1.54
2	Pinch to Hand	0.66
3	Hand to Pinch	1.63
4	Pinch to Hand	1.29
5	Hand to Pinch	1.13
6	Pinch to Hand	1.09
7	Hand to Pinch	0.83
8	Pinch to Hand	0.73
9	Hand to Pinch	0.49
10	Pinch to Hand	0.53
11	Hand to Pinch	1.46
12	Pinch to Hand	0.73
13	Hand to Pinch	0.79
14	Pinch to Hand	1.24
15	Hand to Pinch	0.54
16	Pinch to Hand	1.06
17	Hand to Pinch	1.18
18	Pinch to Hand	0.58
19	Hand to Pinch	1.56
20	Pinch to Hand	2.00
Average Hand to Pinch		1.14
Std. Dev. Hand to Pinch		0.43
Average Pinch to Hand		1.02
Std. Dev. Pinch to Hand		0.45
Overall Average		1.05
Overall Std. Dev.		0.43

**Table 12:** Holding time in the tripod pinch position using a marker-type object.

Trial	Pinch Holding Time (s)
1	10.46
2	5.87
3	19.84
4	12.56
5	13.05
6	22.46
7	19.32
8	26.58
9	20.04
10	24.68
11	22.26
Average pinch holding time	17.92
Standard deviation	6.76

**Table 13:** Results of repeated grip tests performed under controlled initial conditions, indicating successful and failed grasps for different objects.

Marker			Lipstick			Eraser		
Sample	Success	Failure	Sample	Success	Failure	Sample	Success	Failure
1	Yes	No	1	Yes	No	1	Yes	No
2	Yes	No	2	Yes	No	2	Yes	No
3	Yes	No	3	Yes	No	3	Yes	No
4	Yes	No	4	Yes	No	4	No	Yes
5	Yes	No	5	Yes	No	5	Yes	No
6	Yes	No	6	Yes	No	6	Yes	No
7	Yes	No	7	Yes	No	7	Yes	No
8	No	Yes	8	Yes	No	8	No	Yes
9	Yes	No	9	Yes	No	9	Yes	No
10	Yes	No	10	Yes	No	10	Yes	No
11	No	Yes	11	No	Yes	11	Yes	No
12	No	Yes	12	Yes	No	12	Yes	No
13	Yes	No	13	No	Yes	13	Yes	No
14	Yes	No	14	Yes	No	14	Yes	No
15	No	Yes	15	No	Yes	15	No	Yes
16	Yes	No	16	Yes	No	16	Yes	No
17	Yes	No	17	Yes	No	17	Yes	No
18	Yes	No	18	No	Yes	18	Yes	No
19	Yes	No	19	Yes	No	19	Yes	No
20	Yes	No	20	Yes	No	20	No	Yes
Total	16	4	Total	16	4	Total	16	4
Effectiveness	80%		Effectiveness	80%		Effectiveness	80%	

## Data Availability

Data are contained within the article.

## Abbreviations

The following abbreviations are used in this manuscript:

EMG	Electromyography
EEG	Electroencephalography
SSVEP	Visual-evoked potential paradigm
BCI	Brain-Computer Interface
ANFIS	Adaptive Neuro-Fuzzy Inference System
FDM	Fused Deposition Modeling
TPU	Thermoplastic Polyurethane
DIN	Deutsches Institut für Normung
EMA	Exponential moving average
RMS	Root mean square
FCNN	Fully-Connected Feedforward Neural Network
